# Silencing Osa-miR827 via CRISPR/Cas9 protects rice against the blast fungus *Magnaporthe oryzae*

**DOI:** 10.1007/s11103-024-01496-z

**Published:** 2024-09-24

**Authors:** Mireia Bundó, Beatriz Val-Torregrosa, Héctor Martín-Cardoso, María Ribaya, Lidia Campos-Soriano, Marcel Bach-Pages, Tzyy-Jen Chiou, Blanca San Segundo

**Affiliations:** 1 Centre for Research in Agricultural Genomics (CRAG) CSIC-IRTA-UAB-UB. Campus Universitat Autònoma de Barcelona (UAB), Bellaterra (Cerdanyola del Vallés), 08193 Barcelona, Spain; 2 Agricultural Biotechnology Research Center, Academia Sinica No 128, Academia Road, Nankang, Taipei 115 Taiwan; 3 Consejo Superior de Investigaciones Científicas (CSIC), Barcelona, Spain

**Keywords:** Blast, CRISPR/Cas9, *Magnaporthe oryzae*, miR827, Phosphate, *Oryza sativa*

## Abstract

MicroRNAs (miRNAs) are short, non-coding RNAs that regulate gene expression at the post-transcriptional level. In plants, miRNAs participate in diverse developmental processes and adaptive responses to biotic and abiotic stress. MiR827 has long been recognized to be involved in plant responses to phosphate starvation. In rice, the miR827 regulates the expression of *OsSPX-MFS1* and *OsSPX-MFS2,* these genes encoding vacuolar phosphate transporters. In this study, we demonstrated that miR827 plays a role in resistance to infection by the fungus *Magnaporthe oryzae* in rice. We show that *MIR827* overexpression enhances susceptibility to infection by *M. oryzae* which is associated to a weaker induction of defense gene expression during pathogen infection. Conversely, CRISPR/Cas9-induced mutations in the *MIR827* gene completely abolish miR827 production and confer resistance to *M. oryzae* infection. This resistance is accompanied by a reduction of leaf Pi content compared to wild-type plants, whereas Pi levels increase in leaves of the blast-susceptible miR827 overexpressor plants. In wild-type plants, miR827 accumulation in leaves decreases during the biotrophic phase of the infection process. Taken together, our data indicates that silencing *MIR827* confers resistance to *M. oryzae* infection in rice while further supporting interconnections between Pi signaling and immune signaling in plants. Unravelling the role of miR827 during *M. oryzae* infection provides knowledge to improve blast resistance in rice by CRISPR/Cas9-editing of *MIR827.*

**Supplementary Information:**

The online version contains supplementary material available at 10.1007/s11103-024-01496-z.

## Key message

MiR827, a miRNA involved in the control of phosphate homeostasis, is also involved in immunity and disease resistance in rice

**Supplementary Information:**

The online version contains supplementary material available at 10.1007/s11103-024-01496-z.

## Introduction

In natural habitats, plants are simultaneously exposed to combinations of abiotic and biotic stressors that simultaneously or sequentially affect plant growth, yield and overall health (Zandalinas and Mittler 2022). Under such conditions, however, crosstalk between responses induced by a particular type of stress may have an impact on other stresses. Interactions between stressors might have a positive or negative effect on the plant’s response to one or another type of stress (Saijo and Loo 2020). So far, most research on plant responses to environmental stress has been conducted on plants subjected to individual stress.

In agroecosystems, it has long been recognized that nutrient stress caused by deficiency or excess of nutrients (e.g. plants grown in nutrient-poor soils or overfertilized soils, respectively) influences disease resistance (Tripathi et al. 2022). However, differences in disease severity caused by nutritional stress in plants differ depending on the type of nutrient and the identity of the interacting partners, host and pathogen (Veresoglou et al. 2013; Tripathi et al. 2022). Additionally, stress caused by a particular nutrient might have opposite impacts on different diseases, by increasing the incidence of one disease but at the same time decreasing the incidence of other diseases (Tripathi et al. 2022).

When attacked by a pathogen, plants activate multiple defense responses that rely on massive transcriptional reprogramming of gene expression. A two-layer immune system has been defined in plants which depend on the molecules that are recognized by the plant. On the one hand, a general defense response is activated upon recognition of conserved molecular signatures derived from microbes, or Pathogen-Associated Molecular Patterns (PAMPs) by plasma membrane-localized receptors in the host cell, the so-called PAMP-triggered immunity (PTI) (Couto and Zipfel 2016; Jones et al. 2021). PTI involves the activation of multiple signaling pathways leading to the induction of defense-related genes, including *Pathogenesis-Related* (*PR*) genes (Van Loon et al. 2006; dos Santos and Franco 2023). During co-evolution, however, certain pathogens developed mechanisms to inhibit PTI by delivering effectors into the host cell. In turn, plants evolved disease resistance (R) proteins to detect the presence of pathogen effectors, and this recognition triggers signaling events for Effector-triggered immunity (ETI, formerly called gene-for-gene resistance) (Cui et al. 2015; Nguyen et al. 2021). While PTI contributes to resistance to diverse pathogens, ETI is pathogen strain- or race-specific. For a long time, it was considered that PTI and ETI responses to infection by fungal and bacterial pathogens relied on the transcriptional regulation of gene expression. Currently it is also accepted that post-transcriptional regulation of gene expression plays a fundamental role in shaping plant immunity (Song et al. 2021). Indeed, small RNA-mediated gene regulation has emerged as a major mechanism for the control of plant immune responses to pathogen infection, in both PTI and ETI (Bundó et al. 2020; Asadi and Millar 2024).

MicroRNAs (miRNAs) are a class of small RNAs that regulate gene expression post-transcriptionally by base pairing with complementary sequences in the mRNA of target genes (Zhan and Meyers 2023). They are synthesized from *MIR* genes as long precursor transcripts that are processed by a DICER-Like (DCL) ribonuclease through sequential cleavage steps that give rise to miRNA-5p/miRNA-3p duplexes. The functional strand of the mature miRNA duplex is incorporated into the RNA-induced silencing complex (RISC) to direct cleavage or translational inhibition of target transcripts (Llave et al. 2002; Brodersen et al. 2008). *MIR* genes have been categorized into different families based on the mature miRNA and/or precursor sequences. MiRNAs are known to play an important role in executing responses to environmental biotic and abiotic stresses (Millar 2020).

Historically, the functional characterization of miRNAs has been hampered by the lack of loss-of-function mutants. Due to the small size of *MIR* genes, difficulties are encountered in identifying mutant alleles of miRNAs in insertional mutant collections. To overcome this limitation, the use of the CRISPR/Cas9 technology offers the possibility of silencing *MIR* genes and exploration of miRNA function in plants. The CRISPR/Cas9 technology offers numerous possibilities for the functional analysis of miRNAs through the generation of small indels and/or large deletions in the miRNA locus (e.g. by expressing multiple guide RNAs for deletion of the miRNA precursor sequence) (Lowder et al. 2015). CRISPR/Cas9 systems for the transcriptional regulation of gene expression have been also developed, but their applications for the analysis of miRNA function are still in their infancy (Deng et al. 2022).

Rice is one of the world's most important crops and the primary source of food for almost half of its population. Rice production is, however, severely threatened by the blast disease caused by the fungal pathogen *M. oryzae* (Wilson and Talbot 2009; Fernandez and Orth 2018). An important number of miRNAs are known to be responsive to infection by the rice blast fungus *M. oryzae* in rice (Li et al. 2019; Feng et al. 2021; Val-Torregrosa et al. 2021; Javed et al. 2023) yet, the biological functions of most of these pathogen-regulated miRNAs remain elusive. Some examples of miRNAs functionally validated in blast resistance/susceptibility are: miR156fhl, miR160a, miR162a, miR164a, miR167d, miR169a, miR319b, miR396, miR398, miR439, miR444b.2, miR1432, miR7695, miR1873, miR9664, and the polycistronic miRNA miR166k-166 h (Campo et al. 2013, 2021; Li et al. 2014, 2017, 2020, 2021, 2022; Salvador-Guirao et al. 2018; Zhang et al. 2018, 2020; Wang et al. 2018, 2021; Chandran et al. 2019; Bundó et al. 2020; Zhao et al. 2020; Zhou et al. 2020; Lu Junhua et al. 2021; Gao et al. 2023). Depending on the target gene that they regulate, these miRNAs can function as positive or negative regulators of immune responses.

On the other hand, miRNAs also emerged as regulators of plant adaptive responses to nutrient stress (Paul et al. 2015). Most research on miRNAs involved in nutrient homeostasis focused on plants under nutrient-limiting conditions, a situation in which plants improve Pi uptake through the activation of the Phosphate Starvation Response (PSR). Distinct miRNAs are known to be key players in the plant PSR, namely miR399 and miR827. Under Pi deprivation, miR399 and miR827 accumulate (Chien et al. 2018; Paz-Ares et al. 2022; Puga et al. 2024; Yang et al. 2024). In Arabidopsis and rice, miR399 targets *PHO2* (*PHOSPHATE2*), encoding an ubiquitin E2 conjugase implicated in the degradation of the plasma membrane transporter PHT1 (Chiou et al. 2006; Aung et al. 2006; Bari et al. 2006; Liu et al. 2012). As mentioned, the accumulation of miR827 also increases in plants under Pi deprivation conditions (Hsieh et al. 2009; Lin et al. 2010, 2018). Remarkably, miR827 targets different genes in Arabidopsis and rice. In Arabidopsis, miR827 targets *NITROGEN LIMITATION ADAPTATION* (*NLA*) encoding an E3 ubiquitin ligase that mediates the degradation of the plasma membrane transporter PHT1 (Lin et al. 2013). In rice, however, miR827 targets *OsSPX-MFS1* and *OsSPX-MFS2*, these genes encoding SPX-MFS domain-containing proteins that function as vacuolar Pi influx transporters (Lin et al. 2010, 2018; Guo et al. 2023). Vacuoles serve as the primary intracellular compartment for Pi storage and remobilization in plant cells. Accordingly, this organelle plays an important role in the maintenance of cellular Pi homeostasis, a process that highly depends on Pi distribution between vacuoles and cytosol.

Whereas the involvement of miR827 in controlling Pi homeostasis in plants is well documented (Hsieh et al. 2009; Lin et al. 2010; Chien et al. 2017; Paz-Ares et al. 2022; Yang et al. 2024), data obtained by small RNA profiling revealed alterations in miR827 accumulation in rice leaves during infection by *M. oryzae*, or treatment with elicitors prepared from this fungus (Campo et al. 2013; Li et al. 2014, 2016, 2021).

In previous studies, we reported that an increase in Pi content in rice leaves has a negative impact on blast resistance in rice (Campos-Soriano et al. 2020). In this study, we investigated whether, in addition to its role in controlling Pi homeostasis, miR827 also has a function in disease resistance in rice. We show that miR827 overexpression in rice enhances susceptibility to infection by the rice blast fungus *M. oryzae*. Conversely, *MIR827* editing by the CRISPR/Cas9 technology confers resistance to *M. oryzae* infection. Whereas miR827 overexpression is accompanied by an increase in Pi content in rice leaves, miR827 silencing causes a decrease in Pi content. Collectively, these results support that miR827 plays a role in disease resistance in rice and reinforces the notion of interconnections of the molecular mechanisms involved in Pi signaling and immunity in plants.

## Materials and methods

### Plant materials, genotyping and growth conditions

Rice (*Oryza sativa* cv Nipponbare) plants were grown at 28 ºC ± 2 ºC under a 14 h/10 h light/dark cycle. Plants were grown for 21 days in a mixture of 50% peat and vermiculite and 50% quartz sand as substrate and fertilized with modified Hoagland half-strength solution (containing 0.25 mM Pi in control Pi condition, or no Pi added in Low Pi condition) as previously described (Campos-Soriano et al. 2020). Transgenic lines were produced by *Agrobacterium*-mediated transformation of embryogenic *calli* derived from mature rice embryos (Sallaud et al. 2003). The miR827 precursor was constitutively expressed under the control of the maize *ubiquitin* promoter and the *nos* terminator. The production and characterization of miR827 overexpressor plants was previously described (Lin et al. 2010).

### Design of gRNAs and construction of the CRISPR/Cas9 vector for MIR827 editing

For the obtention of CRISPR/Cas9-edited rice plants, we used the system for simultaneous expression of two guide RNAs (gRNAs) described by Lowder et al. (2015). Two gRNAs were designed to target the miR827 precursor sequence (miRBase, Accession No MI0010490) using CRISPR-P 2.0 design tool (http://crispr.hzau.edu.cn/CRISPR2/). The CRISPR-P 2.0 software generates information related to PAM (Protospacer Adjacent Motif) sequences, GC content, number of mismatches, and genome location. In this way, on-target scores were generated for potential gRNAs (scoring 0 to 1, higher score indicating higher efficiency) and also off-target scores for each gRNA (scoring 0 to 1, higher score indicating lower off-target potential). The gRNAs were optimally selected based on the outputs of CRISPR-P 2.0 software.

The selected gRNAs were synthesized as single-stranded oligonucleotides, annealed, cloned into vectors pYPQ131D (gRNA1) and pYPQ132D (gRNA2), and transferred to pYPQ142 vector (upon BsaI digestion and ligation) (Lowder et al. 2015). The expression of gRNAs was driven by the rice U3 RNA polymerase (*OsU3*) promoter (Fig. S1). Finally, pYPQ142 and pYPQ167 (containing the *Cas9* gene) vectors were assembled into pYPQ203 by Gateway recombination (Tang et al. 2017) to obtain the T-DNA binary vector for the generation of CRISPR/Cas9 edited rice plants (Fig. S1). The resulting CRISPR/Cas9 plasmid was used to transform *Agrobacterium tumefaciens* EAH105.

The cultivar Nipponbare (*O. sativa*, *japonica*) was used for rice transformation. CRISPR/Cas 9-edited rice plants were grown under low Pi supply conditions (Campos-Soriano et al. 2020). For genotyping, genomic DNA was obtained from rice leaves using MATAB (0.1 M of Tris–HCl pH 8.0, 1.4 M NaCl, 20 mM EDTA, 2% MATAB, containing 1% PEG 6000 and 0.5% sodium sulfite) as the extraction buffer (Murray and Thompson 1980). PCR was used to amplify the genomic region surrounding the CRISPR/Cas9 target sites. Gene-specific primer pairs were designed using the Primer Blast tool (https://www.ncbi.nlm.nih.gov/tools/primer-blast/). The nucleotide sequences of PCR primers are listed in Table S1. The presence/absence of Cas9 was assessed by PCR. PCR products were analyzed by agarose gel electrophoresis (3% agarose). To identify the exact nature of CRISPR-induced mutations, the PCR products were sequenced.

### Measurement of phosphate content

Free Pi content in rice plants was determined as previously described (Versaw and Harrison 2002). Briefly, rice leaves were frozen in liquid nitrogen and the leaf powder (50 mg) was treated with 1% glacial acid acetic. Ammonium molybdate/ascorbic acid solution (0.7 ml) was then added to the solution. Pi content was calculated by measuring the absorbance at 820 nm.

### Blast resistance assays

The fungus *M. oryzae* (strain Guy 11) was grown on Complete Media Agar (CMA, 9 cm plates, containing 10 mg/L chloramphenicol) for 15 days at 28 °C under a 16 h/8 h light/dark photoperiod condition. Spores were collected by adding sterile water to the mycelium surface and adjusted to the appropriate concentration. Rice plants at the three‐ to four‐leaf stage were spray-inoculated with a spore suspension of *M. oryzae* adjusted at the desired concentration. The percentage of diseased leaf area was determined on the youngest developed leaf (third leaf) of the *M. oryzae*-infected plants at 7 days post-inoculation (dpi) using digital imaging software (APS Assess 2.0 program; Lamari 2008). Fungal biomass was quantified by quantitative PCR (qPCR) using specific primers for the *M. oryzae 28S* DNA gene (Qi and Yang 2002).

### RNA isolation and expression analyses

Total RNA was extracted using TRIzol reagent (Invitrogen). For Northern blot analysis, RNAs (20 µg) were fractionated on denaturing polyacrylamide gels containing 8 M urea, transferred to nylon membranes and probed with [γ^32^P] ATP-labelled oligonucleotides (Table S1).

For RT-qPCR analysis, the first-strand cDNA was synthesized from TURBO DNAse (Ambion) treated total RNA (1 µg) using the High Capacity cDNA reverse transcription kit (Applied Biosystems). Reverse transcription (RT)-qPCR analysis was performed in optical 96-well plates, in a Light Cycler 480, using SYBR® green (Roche). Primers were designed using Primer-Blast (https://www.ncbi.nlm.nih.gov/tools/primer-blast/) (Table S1**)**. The *Ubiquitin1* gene (Os06g0681400) was used to normalize the transcript level in each sample. At least three independent biological replicates (6 pooled leaves per replicate) with three technical replicates were analyzed. Accumulation of mature miR827 was determined by stem-loop reverse transcription quantitative PCR (Varkonyi-Gasic et al. 2007). All the PCR products were confirmed by DNA sequencing. We noticed miR827 sequences currently annotated in miRBase in the miR827 family (miR827 and miR827b; accessions MIMAT0009181 and MIMAT0010075, respectively) are identical sequences, with only a single nucleotide difference at their 5’ terminal position, a typical feature of miRNA isoforms or isomiRs (Fard et al. 2020) (Fig. S2).

## Results

### MIR827 overexpression enhances susceptibility to infection by the rice blast fungus *M. oryzae*

**Fig. 1:**
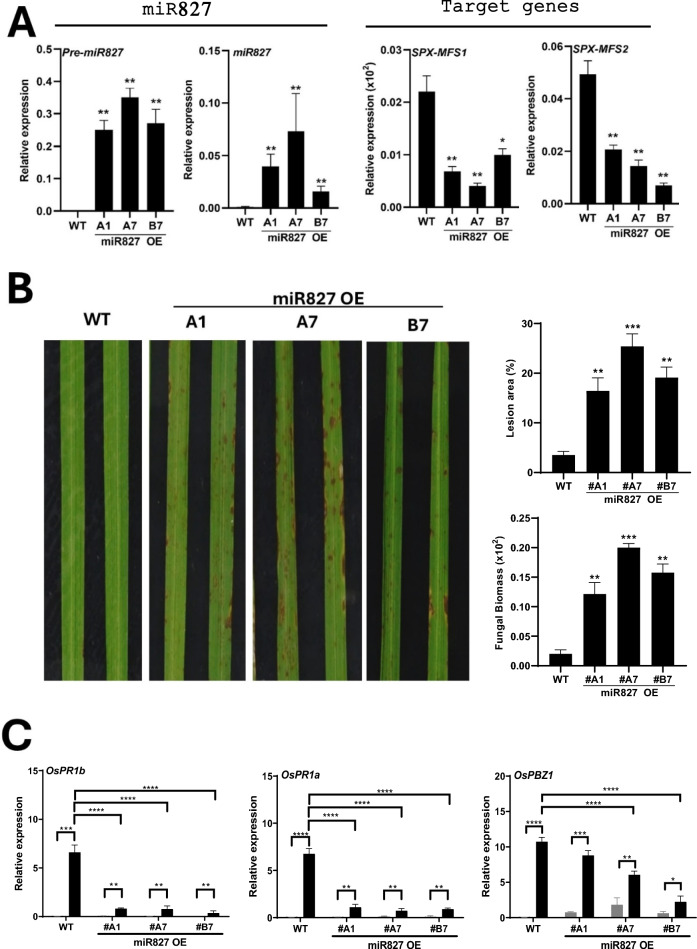
Susceptibility to infection by the rice blast fungus *M. oryzae* in rice plants overexpressing miR827. Bars represent mean ± SEM. Asterisks indicate statistically significant differences calculated by ANOVA (* P ≤ 0.05; ** P ≤ 0.01; *** P ≤ 0.001; **** P ≤ 0.0001). **A** Accumulation of precursor (Pre-miR827) and mature (miR827) miR827 sequences in miR827 OE plants (lines A1, A7, B7) determined by RT-qPCR and stem-loop RT-qPCR, respectively. The expression of miR827 target genes, *OsSPX-MFS1*, *OsSPX-MFS2*, was determined by RT-qPCR. **B** Plants were inoculated with a suspension of *M. oryzae* spore at 5 × 10^5^ spores/ml. Disease symptoms at 7 days post-inoculation (dpi) are shown. Three independent homozygous lines (15 plants in each line) were assayed in each of the four independent experiments with similar results. Right panels, the percentage of the leaf area affected by blast lesions was determined by image analysis. Fungal biomass was quantified by qPCR using specific primers of the *M. oryzae* 28S ribosomal gene relative to the rice *Ubiquitin 1* gene at 7 dpi. Significant differences between each transgenic line and wild-type plants are noted. **C** Accumulation of *OsPR1a*, *OsPR1b* and *OsPBZ1* transcripts in leaves of mock-inoculated (grey bars) and *M. oryzae*-inoculated (black bars) wild-type and miR827 OE plants (48 hpi). Bars represent the mean of three biological replicates (6 pooled leaves in each replicate)

To assess whether miR827 plays a role in disease resistance in rice, we initially assayed blast resistance in rice plants overexpressing *MIR827*. The production and characterization of miR827 overexpressor plants (henceforth miR827 OE) was previously described (Lin et al. 2010). MiR827 accumulation, both precursor and mature sequences, in leaves of miR827 OE plants relative to wild-type plants was confirmed (Fig. 1A, pre-miR827 and miR827, respectively; and Fig. S3A).

In rice, miR827 targets two *OsSPX-MFS* family members, *OsSPX-MFS1* and *OsSPX-MFS2*, encoding vacuolar Pi influx transporters (Lin et al. 2010). As expected, the two miR827 target genes, *OsSPX-MFS1* and *OsSPX-MFS2,* were found to be down-regulated in miR827 OE lines (Fig. 1A).

Resistance to infection by the rice blast fungus *M. oryzae*, was assessed by spray-inoculation with *M. oryzae* spores of soil-grown miR827 OE plants at the three-leaf stage (three independently generated lines) and wild-type plants. At this developmental stage (e.g. three-leaf stage) there were no obvious phenotypic differences between miR827 OE plants and wild-type plants (Fig. S3B). At a later stage of development, however, OE plants exhibited leaf tip necrosis, the typical symptom of Pi toxicity (the same results were previously described; Campo-Soriano et al. 2020).

Importantly, upon inoculation, miR827 OE plants consistently exhibited susceptibility to *M. oryzae* compared with wild-type plants (Fig. 1B, left panel). Blast susceptibility in miR827 OE plants was confirmed by quantifying the leaf area with blast lesions as well as the fungal biomass in the infected leaves (Fig. 1B, right panels). Consistent with the phenotype of blast susceptibility observed in miR827 OE plants, the expression of defense-related genes known to be induced during *M. oryzae* infection (e.g. *OsPR1a*, *OsPR1b* and *OsPBZ1*) was weakly induced in miR827 OE-infected plants compared to wild-type infected plants (Fig. 1C). From these results it is concluded that overexpression of miR827 in rice enhances susceptibility to *M. oryzae* infection.

### CRISPR/Cas9-mediated mutagenesis of MIR827 enhances resistance to infection by M. oryzae

**Fig. 2:**
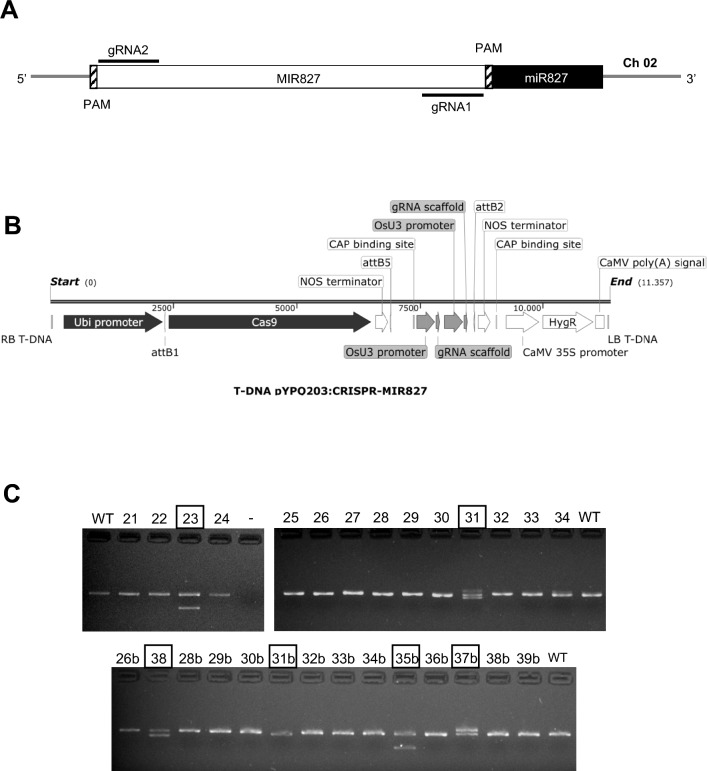
Production and analysis of CRISPR/Cas9-induced mutations in the *MIR827* precursor sequence. **A.** Diagram of the *MIR827* precursor region indicating the location of the two guide RNAs (gRNA1 and gRNA2), the mature miR827 sequence (black box) and PAM sequences (UGG, boxes with dashed lines). **B.** Schematic diagram of components in the T-DNA region of the plasmid used for CRISPR/Cas9 mutagenesis of *MIR827*. The diagram was generated with Snapgene (https://www.snapgene.com). The plasmid named as pYPQ203-MIR827 was used for rice transformation (the steps followed for plasmid preparation are shown in Figure S1). **C.** Genotyping of CRISPR-miR827 lines (T0 generation). Agarose gel electrophoresis of PCR products using the primers spanning the target sites in the miR827 precursor. WT, wild-type allele; -, negative control in the PCR reaction (no template). Boxes indicate selected lines from the T0 generation

To further investigate the function of *MIR827* in disease resistance in rice, we used the CRISPR-P 2.0 genome editing tool (http://crispr.hzau.edu.cn/CRISPR2/) for designing specific gRNAs for CRISPR/Cas9-directed mutagenesis in the *MIR827* locus (Liu et al. 2017). Two PAM (protospacer adjacent motif, NGG) sequences were present in the miR827 precursor sequence, one of them being adjacent to the mature miR827 sequence and the other one at the 5’ end of the miR827 precursor sequence registered in miRBase (Fig. 2A). Based on criteria established for gRNA design by the CRISPR-P2.0 software, two guide RNAs (gRNA1 and gRNA2) potentially capable of directing mutations at the *MIR827* locus were identified. The nucleotide sequences for target sites of gRNAs were as follows (in bold, PAM sequences): 5’-TATTGGCTCTTGGGCACGCG**TGG**-3’, for gRNA1; 5’-TAGATGACCAGCAACAAAAC**AGG**-3’, for gRNA2. The binary vector for rice transformation contained the *Cas9* gene and the two guide RNAs (Fig. 2B). Transgenic rice (*O. sativa* cv Nipponbare) plants were produced by *Agrobacterium*-mediated transformation of embryonic *calli* derived from mature embryos (Sallaud et al. 2003). A total of 48 independent T0 hygromycin-resistant lines were obtained.

CRISPR/Cas9-mediated mutations were identified using the polymerase chain reaction (PCR) followed by agarose gel electrophoresis and amplicon sequencing. PCR products obtained from these lines (T0 generation) showed a different pattern in comparison to the wild-type (Fig. 2C). Upon DNA sequencing of PCR products, the CRISPR/Cas9-induced mutations were identified using the web-based application CRISPR-ID (http://crispid.gbiomed.kuleuven.be/).

**Fig. 3:**
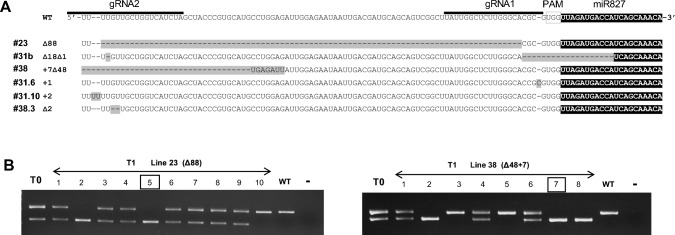
Mutations generated in the *MIR827* precursor sequence (T1 generation). **A.** CRISPR/Cas9-induced mutations are highlighted in grey. Dashes denote deletions. **B.** Agarose gel electrophoresisof PCR products. Boxes indicate selected CRISPR-edited lines harboring a deletion of 88 nucleotides (Δ88, Line 23.5), or a deletion of 48 nucleotides and an insertion of 7 nucleotides (Δ48/ + 7, Line 38.7)

CRISPR/Cas9-induced mutations consisted in small indels (1 or 2 nucleotides), and large deletions in the miRNA precursor (Fig. 3A). We reasoned that large deletions on the miR827 precursor are likely to disrupt the precursor structure, thus preventing miRNA precursor processing and production of mature miR827 sequences. Indeed, prediction of the secondary structure of the wild-type and CRISPR/Cas9-edited mutant alleles indicated that mutant alleles containing large deletions have lost their capability to fold into a stem-loop structure (Fig. S4A; lines 23 and 38). This analysis also revealed that the mature miR827 sequence mapped to the 3’ end of the stem region in the precursor structure (based on the precursor sequence currently annotated in miRBase). Clearly, this feature does not meet the requirements for miRNA precursor processing, as longer structures are required for excision of the mature miRNA from the precursor (e.g. lower stem region is not present in the miR827 precursor structure). By extending the nucleotide sequence of the genomic region surrounding the *MIR827* locus (20 nucleotides upstream and downstream), a stem-loop hairpin structure could be predicted in which the mature miR827 sequence maps within a longer stem region (Fig. S4B).

Mutant lines containing large deletions were then used for the obtention of homozygous plants for the mutation in the miR827 locus. They were: line 23 (deletion of 88 nucleotides, ∆88) and line 38 (deletion of 48 nucleotides and insertion of 7 nucleotides, ∆48/ + 7). Homozygous lines for each mutation (e.g. lines 23.5 and 38.7) were identified in the T1 generation and further analyzed (hereinafter named as CRISPR-miR827 lines) (Fig. 3B).

**Fig. 4:**
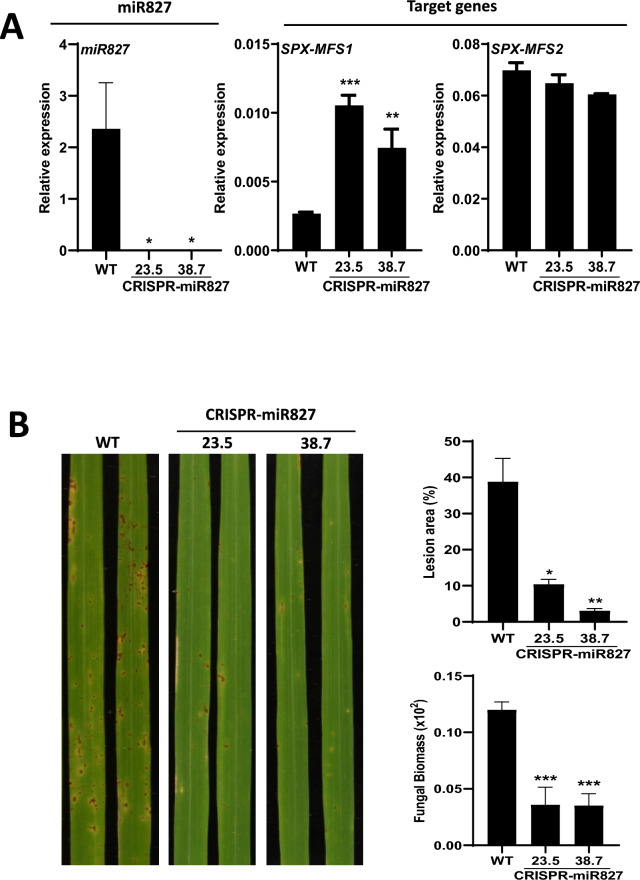
CRISPR/Cas9- induced mutations in the *MIR827* gene confer resistance to *M. oryzae* infection. Statistical significance was determined by ANOVA (* P ≤ 0.05, ** P ≤ 0.01, *** P ≤ 0.001). **A** Accumulation of mature miR827 sequences was determined by stem-loop RT-qPCR (left panel). Transcript levels of miR827 target genes, *OsSPX-MFS1* and *OsSPX-MFS2* were measured by RT-qPCR (middle and right panels). *OsSPX-MFS2* expression was not significantly affected in the CRISPR-miR827 lines compared to wild-type plants. **B** Plants were inoculated with a suspension of *M. oryzae* spore at 1 × 10^6^ spores/ml. Representative images of *M. oryzae*-infected leaves of CRISPR-miR827 and WT plants at 5 days post-inoculation are shown. The percentage of diseased area was determined by image analysis. Quantification of fungal biomass was performed by qPCR using specific primers for *M. oryzae* 28S DNA normalized against the rice *Ubiquitin 1* gene was used to quantify fungal biomass. Results are from one out of three independent experiments (8 plants/genotype) which gave similar results

Interestingly, mature miR827 accumulation was completely abolished in CRISPR-miR827 plants, thus, supporting effective silencing of *MIR827* expression (Fig. 4A, left panel). This is consistent with up-regulation of *OsSPX-MFS1* expression in CRISPR-miR827 plants (Fig. 4A, middle panel). Intriguingly, the expression of *OsSPX-MFS2* (also a target gene for miR827) was not significantly altered in CRISPR-miR827 plants (Fig. 4A, right panel). Here, it is worth mentioning that complex regulatory mechanisms have been proposed to occur in wild-type plants for the control of *OsSPX-MFS2* expression (Lin et al. 2010). Along with this, previous studies in Pi-starved wild-type plants revealed up-regulation of *MIR827* and down-regulation of *OsSPX-MFS1*, while *OsSPX-MFS2* expression was found to be up-regulated in Pi-starved wild-type plants. Still unknown mechanisms (e.g. mechanisms other than miR827-guided cleavage of *OsSPX-MFS2* transcripts) must then operate for the control of *OsSPX-MFS2* expression in rice, an aspect that deserves further investigation.

For blast resistance assays, CRISPR-miR827 (homozygous 23.5 and 38.7 plants) and wild-type rice plants were grown under low Pi supply, a condition in which miR827 is known to accumulate in wild-type plants. Blast resistance was assayed in plants at the 3–4 leaf stage. Up-regulation of *OsPHR4* encoding a Pi starvation-inducible rice gene (Ruan et al. 2017) confirmed that rice plants perceive and respond to low Pi treatment (Fig. S5B). At this developmental stage, no phenotypic differences were observed between wild-type and CRISPR-miR827 lines grown either under low-Pi supply or sufficient Pi conditions (Fig. S5A). At a later developmental stage, however, the CRISPR-miR827 plants showed reduced growth compared with wild-type plants. Importantly, CRISPR-miR827 plants showed resistance to *M. oryzae* infection as revealed by visual inspection of infected plants, quantification of diseased leaf area and measurement of fungal biomass (Fig. 4B).

The rice *SPX-MFS* family comprises three members which encode vacuolar Pi transporters, namely *OsSPX-MFS1*, *OsSPX-MFS2,* and *OsSPX-MFS3*. Of them, *OsSPX-MFS1* and *Os-SPX-MFS2*, but not *OsSPX-MFS3*, are under regulation by miR827 (Lin et al. 2018; Zhang et al. 2020). The observation that *OsSPX-MFS3* expression is not altered in CRISPR-miR827 plants (Fig. S5C) excluded the possibility of compensatory mechanisms affecting *OsSPX-MFS3* expression caused by *MIR827* silencing.

Finally, we carried out a search for potential off-target sites for each gRNAs used to create deletion in the *MIR827* locus. Potential off-target sites were investigated using the CRISPR-P 2.0 tool (http://crispr.hzau.edu.cn/CRISPR2/; Liu et al. 2017) and their scores were calculated according to the algorithm described by Doench et al. (2016). The occurrence of off-target modifications depends on the location of the gRNA and genomic DNA pairings as well as on the Cas9’s capacity to function (Cas9 is known to tolerate up to 3 mismatches between gRNA and genomic DNA) (Sturme et al. 2022). For instance, off-target effects are reduced when the mismatches are located within the first 8–10 nucleotides proximal to the PAM known, the seed region, which is critical for binding the gRNA-Cas9 nuclease complex to the target sequence (Modrzejewski et al. 2020). On these bases, potential off-target candidates were identified for each gRNA (Table S2). For gRNA1, only 1 sequence was predicted as a potential off-target with 3 mismatches, which locates in an intergenic region (Table S2). For gRNA2, a potential target was predicted at the untranslated region of LOC_Os01g55240, with 3 mismatches. One of these mismatches, however, is located on the seed region, upstream of the PAM site. This locus encodes a gibberellin 2-beta-dioxygenase (*OsGA2ox3*) involved in seed vigor (Table S2). Although *OsGA2ox3* expression has not been examined in CRISPR-miR827 plants, no differences in seed vigor could be observed between CRISPR-miR827 and wild-type plants.

Altogether, this study demonstrated that silencing *MIR827* expression confers resistance to infection by the blast fungus *M. oryzae,* which is consistent with the observed phenotype of blast susceptibility in miR827 OE plants. These findings also illustrate the usefulness of the CRISPR/Cas9 genome editing system for functional analyses of *MIR* genes. Presumably, deletions generated by CRISPR/Cas9 impair the processing of the miR827 precursor and prevent the accumulation of mature miR827 species for enhanced rice blast resistance.

### miR827 expression is down-regulated during fungal infection

**Fig. 5:**
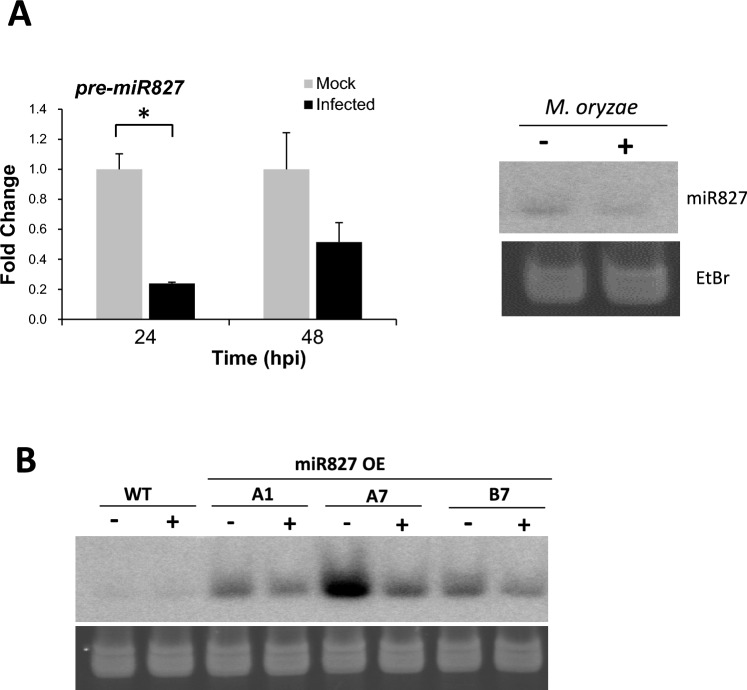
Accumulation of precursor (pre-miR827) and mature miR827 sequences during infection of wild-type (WT) and miR827 OE plants with *M. oryzae*. **A** Accumulation of miR827 precursor transcripts in mock-inoculated and *M. oryzae*-inoculated wild-type plants (- and + , respectively) at 24 and 48 hours post-inoculation (hpi) (left panel). RT-qPCR analysis was carried out using the rice *Ubiquitin 1* gene as the internal control. The expression level in mock-inoculated plants was set to 1.0. Right panel, accumulation of mature miR827 sequences in mock-inoculated and *M. oryzae*-inoculated, plants (- and + , respectively) at 24 hpi determined by Northern blot analysis. **B** Northern blot analysis of small RNAs obtained from miR827 OE plants. RNAs were probed with synthetic oligonucleotides complementary to miR827 (upper panels). Lower panels, ethidium bromide staining of RNAs

Having established that alterations in *MIR827* expression have an impact on blast resistance, we investigated whether *MIR827* expression itself is regulated during *M. oryzae* infection. For this, wild-type rice plants (cv. Nipponbare) were grown for 3 weeks under low-Pi conditions (a condition in which miR827 accumulates in wild-type plants), and then inoculated with *M. oryzae* spores, or mock-inoculated. Compared to mock-inoculated plants, the accumulation of miR827 precursor transcripts decreased early during infection in wild-type rice plants (this reduction in miR827 precursor levels was more evident at 24 hpi) (Fig. 5A, left panel). Consistent with a reduction in miR827 precursor transcripts, the accumulation of mature miR827 sequences was also reduced in response to *M. oryzae* infection in wild-type plants (Fig. 5A, right panel). Finally, pathogen-inducible suppression on miR827 accumulation was also observed during infection of miR827 OE plants (Fig. 5B). Taken together, these results indicated that fungal infection negatively regulates *MIR827* expression.

### Alterations in *MIR827* expression result in modified Pi levels

**Fig. 6:**
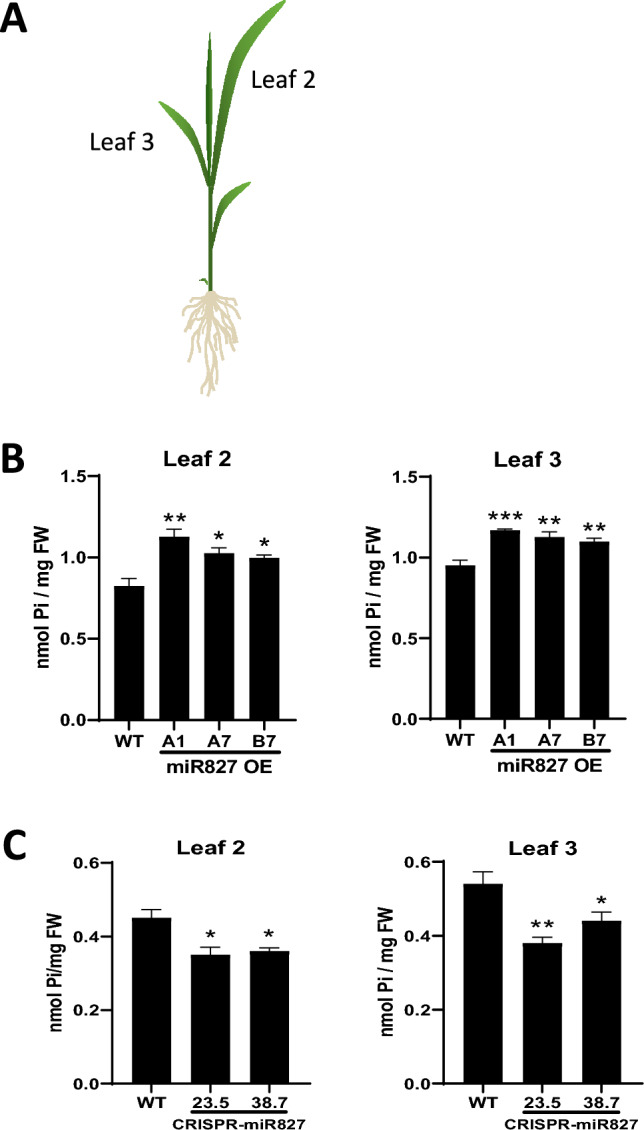
Pi content in leaves of miR827 OE and CRISPR-miR827 plants. MiR827 OE plants were grown under sufficient Pi conditions and CRISPR-miR827 plants under low-Pi conditions. **A.** Pi content was assessed in leaves at two different positions of the same plant, Leaf 2 and Leaf 3. **B.** Pi content in leaves of miR827 OE plants. **C.** Pi content in CRISPR-miR827 plants. Three independent experiments were conducted with similar results. Values shown are the mean ± SEM of four biological replicates (leaves from 6 plants per replicate). Asterisks denote statistical significance relative to wild-type plants (t-test, * P ≤ 0.05, ** P ≤ 0.01, *** P ≤ 0.001)

To establish a relationship between Pi content and blast resistance, we measured Pi content in miR827 OE and CRISPR-miR827 plants. Measurement of Pi content was carried out in two different leaves of the rice plants, namely leaves at positions 2 and 3, leaf 1 corresponding to the youngest fully developed leaf. Compared with WT plants, miR827 OE plants had a higher Pi content in both leaves (Fig. 6). Conversely, *MIR827* silencing in CRISPR-miR827 plants is accompanied by a reduction in Pi content (Fig. 6). In previous studies, we reported that high Pi fertilization and subsequent accumulation of Pi in rice leaves increases susceptibility to *M. oryzae* infection (Campos-Soriano et al. 2020). On this basis, increased Pi levels in miR827 OE plants might also contribute to the observed phenotype of blast susceptibility in these plants. A decrease in Pi content in the CRISPR-miR827 plants relative to wild-type plants would also explain increased blast resistance in miR827-silenced plants. Finally, pathogen infection does not provoke alterations in Pi content either on miR827 OE plants or CRISPR-miR827 plants (Fig. S6).

Altogether, these findings support that an alteration in *MIR827* expression provokes changes in Pi content in leaves of rice plants, and that these alterations might contribute to a phenotype of disease resistance, that is, susceptibility in miR827 overexpressor and resistance in miR827-silenced plants.

## Discussion

Advances in the identification of rice miRNAs during the past years have demonstrated that an important number of miRNAs are responsive to infection by *M. oryzae*, or treatment with *M. oryzae* elicitors (Campo et al. 2013; Li et al. 2014, 2016, 2021; Baldrich et al. 2015). However, the biological function of most of these pathogen-regulated miRNAs in rice immunity needs further understanding. Among those *M. oryzae*-regulated rice miRNAs identified through small RNA sequencing data was miR827, a miRNA which has long been demonstrated for its role in regulating Pi homeostasis.

Here we show that, in addition to Pi homeostasis, miR827 plays a negative role in disease resistance in rice. Supporting this conclusion, CRISPR/Cas9-mediated silencing of *MIR827* expression increased blast resistance, while miR827 overexpression enhanced susceptibility. An alteration in *MIR827* expression was accompanied by changes in Pi content, with increased and decreased free Pi content in miR827OE and CRISPR/Cas9 plants, respectively. Previous studies demonstrated that Pi accumulation in rice leaves repressed *M. oryzae*-inducible *PR* expression, hence, increasing blast susceptibility, a situation referred to as Pi-induced susceptibility (Campos-Soriano et al. 2020; Martín-Cardoso et al. 2024). Along with this, a weaker induction of *OsPR1* and *OsPBZ1* expression occurs in miR827 OE plants during *M. oryzae* infection which correlates well with the phenotype of blast susceptibility observed in these plants. Overall, results obtained in this study support the conclusion that miR827 functions as a negative regulator of rice immunity.

A further consideration relates to the function of miRNAs as regulators of gene expression in plants. Dynamic changes are known to occur in the expression of *MIR* genes supporting the notion that miRNAs serve to fine-tune gene expression rather than turning-on or turning-off target gene expression. Indeed, small RNA sequencing data obtained from *M. oryzae*-infected and/or elicitor-treated rice leaves indicated that miR827 accumulation varies over time during the infection process, with changes not only in miR827 levels but also in the trend of regulation (up- and down-regulation) (Campo et al. 2013; Li et al. 2016, 2021). MiRNAs are then perfectly suited for dynamic regulation of gene expression when plants are exposed to combinations of stressors simultaneously. Results presented in this study demonstrate that miR827 mediates crosstalk between Pi-induced signaling pathways and pathogen-induced signaling pathways in rice.

As previously mentioned, the rice SPX-MFS family consists of three genes (*OsSPX-MFS1*, *OsSPX-MFS2* and *OsSPX-MFS3*) encoding vacuolar Pi transporters. Of them, *OsSPX-MFS1* and *OsSPX-MFS2* are regulated by miR827. Recently, the three rice SPX-MFS proteins were reported to act as vacuolar Pi influx transporters, although they are not functionally equivalent (Guo et al. 2023). Pi efflux transporters have been also described in rice, such as *OsVPE1* and *OsVPE2* (Vacuolar Pi Efflux transporters 1 and 2), (Xu et al. 2019). Clearly, Pi distribution between the vacuole and the cytoplasm must be maintained within a relatively narrow but dynamic range to ensure optimal intracellular Pi status under variable Pi supply. It is then tempting to hypothesize that miR827-mediated regulation of *OsSPX-MFS1* and *OsSPX-MFS2*, may be an important factor in controlling the intracellular Pi status (e.g. vacuolar/cytoplasmic distribution) and that alterations in vacuolar/cytosolic Pi distribution might be involved in blast resistance in rice. Before concluding this, a better knowledge of the relative contribution of rice vacuolar Pi transporters in controlling cytoplasmic/vacuolar distribution is still needed. This will allow us to better understand the impact of miR827-mediated regulation in the expression of vacuolar Pi transporters (*OsSPX-MFS1* and *OsSPX-MFS2*) and Pi homeostasis in rice plants.

Being a foliar pathogen, *M. oryzae* has an absolute requirement for Pi from the host tissue. This fungus has a hemibiotrophic lifestyle consisting of an initial biotrophic phase in which the fungus invades living host cells and acquires nutrients from living host cells, followed by a necrotrophic phase in which the fungus promotes host cell death and acquires nutrients from dead or dying cells (Fernandez and Orth 2018). Studies in the rice/*M. oryzae* interactions also demonstrated that host vacuole integrity in invaded cells needs to be maintained until a certain stage during the biotrophic invasion, a period in which the rice vacuole progressively shrinks and eventually is disrupted (Mochizuki et al. 2015). Vacuole disruption coincides with the death of the invaded cell and marks the end of biotrophy (Jones et al. 2021). Only disruption of the host vacuole during necrotrophy would provide a nutrient-rich environment to the fungus to facilitate host tissue invasion. In this work, we found that *MIR827* expression is down-regulated during the biotrophic stage of *M. oryzae* infection (e.g. up to 48 hpi). The observed reduction in miR827 accumulation during biotrophy is expected to be accompanied by up-regulation of the miR827 target genes (at least *OsSPX-MFS1*) and subsequent increase in SPX-MFS protein levels. If so, this regulatory mechanism would favor Pi influx into the vacuole. Vacuolar sequestration of Pi away from the invading pathogen during biotrophy would then restrict fungal growth. Based on these considerations, temporal changes in *MIR827* expression might well modulate the amount of Pi available for fungal growth and host cell colonization during biotrophy. In this work, *miR827* expression was not examined at a later stage of infection (e.g. later than 48 hpi), a period in which host cell lysis would occur. MiR827-mediated regulated processes controlling cellular Pi homeostasis might then be a factor in determining resistance or susceptibility to the rice blast fungus *M. oryzae* in rice.

In addition to the lifestyle of the pathogen (e.g. necrotrophic, hemibiotrophic and biotrophic lifestyle), the impact of host Pi content in disease resistance also appears to be dependent on the plant species. While Pi accumulation increases susceptibility to infection by *M. oryzae* in rice, its accumulation in Arabidopsis confers resistance to infection by necrotrophic (*Plectosphaerella cucumerina*) and hemibiotrophic (*Colletotrichum higginsianum*) fungal pathogens (Val-Torregrosa et al. 2022b). Moreover, *MIR827* overexpression and loss-of-function of *NLA* (the target gene of miR827 in Arabidopsis) were reported to increase Pi level and to enhance resistance to *P. cucumerina* in Arabidopsis (Val-Torregrosa et al. 2022a). Therefore, it will be of interest to determine the impact of Pi content in rice plants during interaction with other types of pathogens, an aspect that deserves further investigation.

On the other hand, it is well known that miR827 has different target genes in rice and Arabidopsis. In Arabidopsis, miR827 targets *NLA* which encodes an E3 ubiquitin ligase that mediates the degradation of the plasma membrane transporter PHT1, whereas rice miR827 targets the two vacuolar Pi transporters, *OsSPX-MFS1* and *OsSPX-MFS2* (Lin et al. 2010, 2013). A transition of miR827 target preference during plant evolution has been described (Lin et al. 2018). Despite the evolution of miR827 targeting, upregulation of *MIR827* by Pi starvation for the control of Pi homeostasis has been retained during evolution. As different strategies appear to be used in different plant species for the control of cellular Pi homeostasis, the functioning of the miR827/target gene pair(s) in each plant species might be responsible for different resistance phenotypes (e.g. susceptibility to pathogen infection in rice, but resistance in Arabidopsis). The possibility that miR827 has evolved the ability to regulate target genes other than *OsSPX-MFS* genes in rice should be also considered. In this respect, studies in Arabidopsis identified *GPLα*, a member of the Glabrous-enhancer-binding protein family of transcription factors, as a possible target of miR827 (Ma et al. 2021). The effect of Pi on disease resistance might then be dependent on specificities in Pi-induced signaling pathways, and/or miR827 target preference, in different plant species.

Collectively, the results here presented in rice, together with those previously reported in other pathosystems (Castrillo et al. 2017; Luo et al. 2021; Chan et al. 2021; Dindas et al. 2022), illustrate the relevance of Pi in controlling disease resistance in plants while reinforcing the notion of cross-talk between Pi signaling and immune signaling in plants. From a practical point of view, these findings have important implications for crop production. Even though total Pi in soils is relatively abundant, its bioavailability is very low due to the large reactivity of Pi with numerous soil components*.* To solve this problem, modern agriculture is highly dependent on the use of P fertilizers which causes eutrophication of surface water systems and has a negative impact on animal health and the environment. On the other hand, *M. oryzae* is responsible for the rice blast disease, one of the most devastating fungal diseases of rice worldwide, and agrochemicals are widely used for blast control in rice cultivation. Paradoxically, excessive use of Pi fertilizers might enhance susceptibility to the rice blast fungus, thus, increasing dependence on pesticides for rice production. To be environmentally safe, new strategies need to be developed for sustainable rice production by reducing dependence on Pi fertilizers and pesticides. In this direction, a better knowledge of cross-talk between Pi signaling pathways and immune responses will lay a foundation for the rational use of fertilizers and pesticides which will also help design novel strategies to improve blast resistance in rice.

## Supplementary Information

Below is the link to the electronic supplementary material.

Supplementary file1 (PPTX 49 KB) Strategy followed for construction of the plasmid used for CRISPR/Cas9-based genome editing of MIR827 in rice. The gRNA1 and gRNA2 were introduced into vectors pYPQ131D and pYPQ132D, respectively (Lowder et al., 2015). OsU3, rice U3 RNA polymerase promoter; ZmUbi, maize Ubiquitin 1 promoter; T, transcription terminator. "att" indicates gateway recombination sites

Supplementary file2 (DOCX 13 KB) Alignment of mature miR827 sequences registered in miRBase named as miR827 and miR827b (MIMAT009181 and MIMAT0010075, respectively). The two small RNAs, miR827 and miR827b, have the same nucleotide sequence, only differing in 1 nucleotide at the 5’ end, thus, corresponding to isomiRNAs for miR827

Supplementary file3 (PPTX 887 KB) Characterization of miR827 OE rice plants. Wild-type (WT) and miR827 OE plants (independent homozygous lines A1, A7 and B7) were grown for 3 weeks under greenhouse conditions. A. Northern blot analysis of RNAs obtained from leaves of miR827 OE and wild-type plants. RNAs were probed with [γ32P] ATP-labelled oligonucleotides complementary to the miR827 sequence. Lower panel shows RNAs stained with ethidium bromide. B. Appearance of 3-week-old wild-type and miR827 OE plants

Supplementary file4 (PPTX 396 KB) Predicted secondary structure of the miR827 precursor, wild-type (WT) and mutant alleles generated by CRISPR/Cas9 mutagenesis. The fold-back structure of the mutated miR827 precursor was obtained using the RNAfold Web Server (http://rna.tbi.univie.ac.at/cgi-bin/RNAWebSuite/RNAfold.cgihttp://rna.tbi.univie.ac.at/cgi-bin/RNAWebSuite/RNAfold.cgi). The location of the mature miR827 in the precursor structure is indicated with a black line. Colors indicate the base pairing probability. A. Predicted precursor structure of the wild-type and mutant miR827 precursors based on the nucleotide sequence currently annotated in miRBase. B. Precursor structure predicted for the wild-type miR827 precursor upon extending its nucleotide sequence from the genomic region surrounding the MIR827 locus (20 nucleotides upstream/downstream)

Supplementary file5 (PPTX 660 KB) Appearance of CRISPR-miR827 rice plants and expression analysis of *OsPHR4* and *OsSPX-MFS3* in CRISPR-miR827 plants. A. Phenotype of CRISPR-miR827 (lines 23.5 and 38.7) and wild-type (WT, e.g. azygous plants segregated from heterozygous plants) plants. Rice plants were grown under Pi-limiting conditions (Low-Pi) or Pi sufficient conditions for 3 weeks. B. *OsPHR4* expression in wild-type and CRISPR-miR827 rice plants grown under Low-Pi supply (black bars) and normal (white bars) Pi conditions. C. *OsSPX-MFS3* expression in CRISPR-miR827 plants was determined by RT-qPCR. Data from one representative experiment of three independent experiments are presented, each experiment consisting of a pool of 3 leaves (ANOVA test, * P ≤ 0.05; ** P ≤ 0.01; *** P ≤ 0.001; ns, not significant)

Supplementary file6 (PPTX 70 KB) Pi content in leaves of wild-type, miR827 OE (upper panel) and CRISPR-miR827 (lower panel) plants that have been mock-inoculated (-) or inoculated with *M. oryzae* spores (+). Pi content was assessed in leaves at two different positions of the same plant, Leaf 2 and Leaf 3, at 24 hours post-inoculation (hpi). Results shown correspond to Leaf 2 (similar results were obtained on leaves at position 3). Bars represent mean ± SEM of 4 biological replicates, each one from a pool of 4 leaves (leaf 2) from independent plants (Two-way ANOVA followed by Tukey’s HSD test). Letters indicate significant differences among conditions

Supplementary file7 (XLSX 15 KB) Oligonucleotides used in this study

Supplementary file8 (DOCX 19 KB) Prediction of off-targets for gRNAs used to generate mutations in the MIR827 locus. The CRISPR-P 2.0 software designed for genome editing was used for off-target prediction (http://crispr.hzau.edu.cn/CRISPR2/). The scoring system in the CRISPR-P 2.0 tool identifies the most similar sequences with a limited number of mismatches (typically ≤ 3) in comparison to the designed gRNA. The off-target score ranges from 0 to 1, with a higher score denoting targets with lower target potential. The top 20 predicted off-targets are shown. PAM sequences are highlighted in green. Mismatches are indicated in red

## Data Availability

All data generated or analysed during this study are included in this published article and its supplementary information files.
