# Supplementary material for: Silencing Osa-miR827 via CRISPR/Cas9 protects rice against the blast fungus Magnaporthe oryzae
Source: Plant Mol Biol. 2024 Sep 24;114(5):105. doi: 10.1007/s11103-024-01496-z (PMC11422438; doi:10.1007/s11103-024-01496-z)
Supplement: Supplementary file 1 — Supplementary file1 (PPTX 49 KB) Strategy followed for construction of the plasmid used for CRISPR/Cas9-based genome editing of MIR827 in rice. The gRNA1 and gRNA2 were introduced into vectors pYPQ131D and pYPQ132D, respectively (Lowder et al., 2015). OsU3, rice U3 RNA polymerase promoter; ZmUbi, maize Ubiquitin 1 promoter; T, transcription terminator. "att" indicates gateway recombination sites [file 11103_2024_1496_MOESM1_ESM.pptx]

## Slide 1
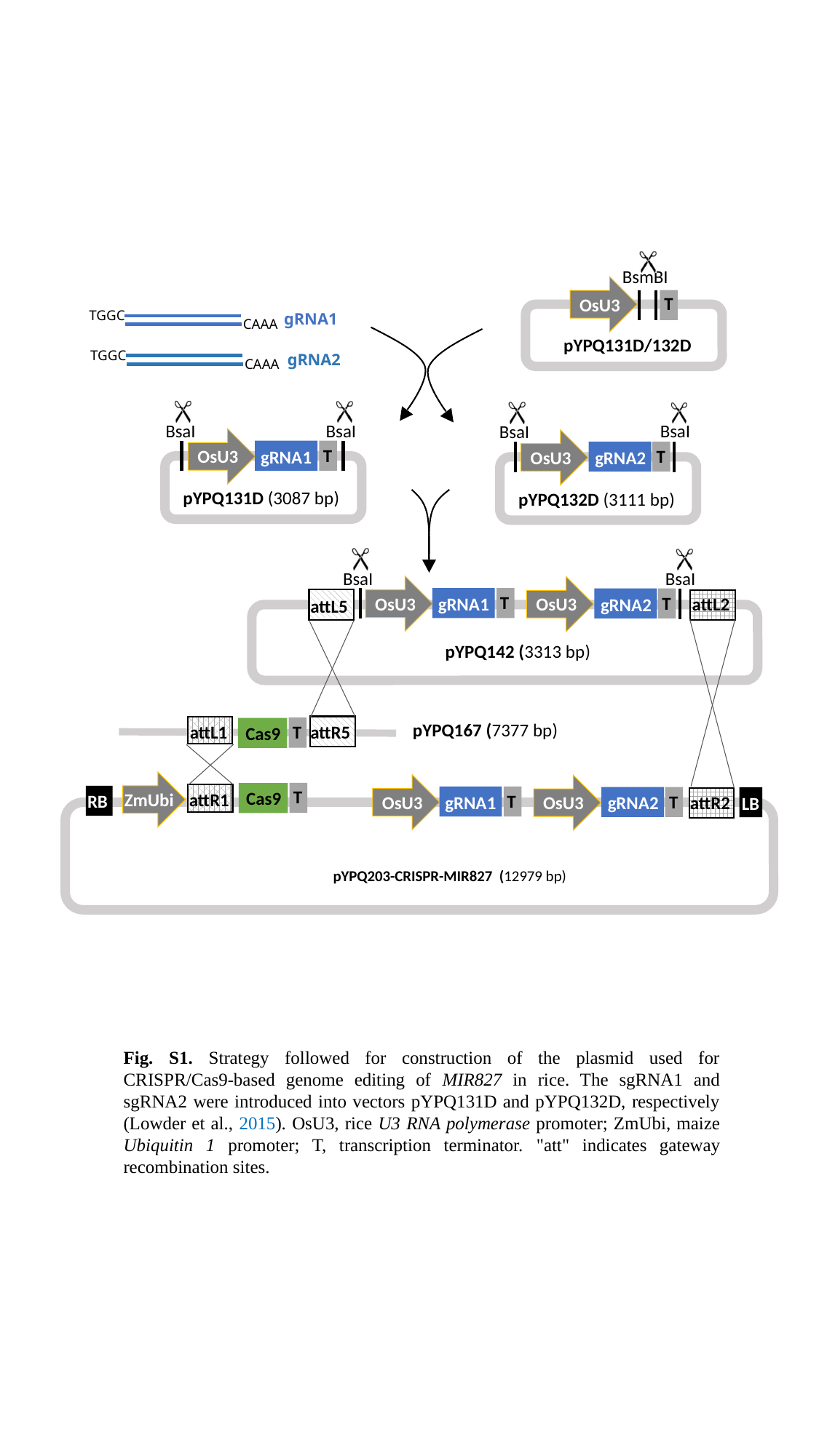

BsmBI
T
OsU3
pYPQ131D/132D
TGGC
gRNA1
CAAA
TGGC
gRNA2
CAAA
BsaI
BsaI
BsaI
BsaI
T
T
OsU3
gRNA1
OsU3
gRNA2
pYPQ131D (3087 bp)
pYPQ132D (3111 bp)
BsaI
BsaI
T
T
OsU3
gRNA1
attL2
OsU3
gRNA2
attL5
pYPQ142 (3313 bp)
pYPQ167 (7377 bp)
T
attL1
attR5
Cas9
T
Cas9
ZmUbi
attR1
T
RB
T
OsU3
gRNA1
OsU3
gRNA2
attR2
LB
pYPQ203-CRISPR-MIR827 (12979 bp)
Fig. S1. Strategy followed for construction of the plasmid used for CRISPR/Cas9-based genome editing of MIR827 in rice. The sgRNA1 and sgRNA2 were introduced into vectors pYPQ131D and pYPQ132D, respectively (Lowder et al., 2015). OsU3, rice U3 RNA polymerase promoter; ZmUbi, maize Ubiquitin 1 promoter; T, transcription terminator. "att" indicates gateway recombination sites.
