# Supplementary material for: Silencing Osa-miR827 via CRISPR/Cas9 protects rice against the blast fungus Magnaporthe oryzae
Source: Plant Mol Biol. 2024 Sep 24;114(5):105. doi: 10.1007/s11103-024-01496-z (PMC11422438; doi:10.1007/s11103-024-01496-z)
Supplement: Supplementary file 2 — Supplementary file2 (DOCX 13 KB) Alignment of mature miR827 sequences registered in miRBase named as miR827 and miR827b (MIMAT009181 and MIMAT0010075, respectively). The two small RNAs, miR827 and miR827b, have the same nucleotide sequence, only differing in 1 nucleotide at the 5’ end, thus, corresponding to isomiRNAs for miR827 [file 11103_2024_1496_MOESM2_ESM.docx]

**osa-miR827**  -UUAGAUGACCAUCAGCAAACA 21 ntd

**osa-miR827b** GUUAGAUGACCAUCAGCAAAC- 21 ntd

************************

**Figure S2**
