# Supplementary material for: Silencing Osa-miR827 via CRISPR/Cas9 protects rice against the blast fungus Magnaporthe oryzae
Source: Plant Mol Biol. 2024 Sep 24;114(5):105. doi: 10.1007/s11103-024-01496-z (PMC11422438; doi:10.1007/s11103-024-01496-z)
Supplement: Supplementary file 3 — Supplementary file3 (PPTX 887 KB) Characterization of miR827 OE rice plants. Wild-type (WT) and miR827 OE plants (independent homozygous lines A1, A7 and B7) were grown for 3 weeks under greenhouse conditions. A. Northern blot analysis of RNAs obtained from leaves of miR827 OE and wild-type plants. RNAs were probed with [γ32P] ATP-labelled oligonucleotides complementary to the miR827 sequence. Lower panel shows RNAs stained with ethidium bromide. B. Appearance of 3-week-old wild-type and miR827 OE plants [file 11103_2024_1496_MOESM3_ESM.pptx]

## Slide 1
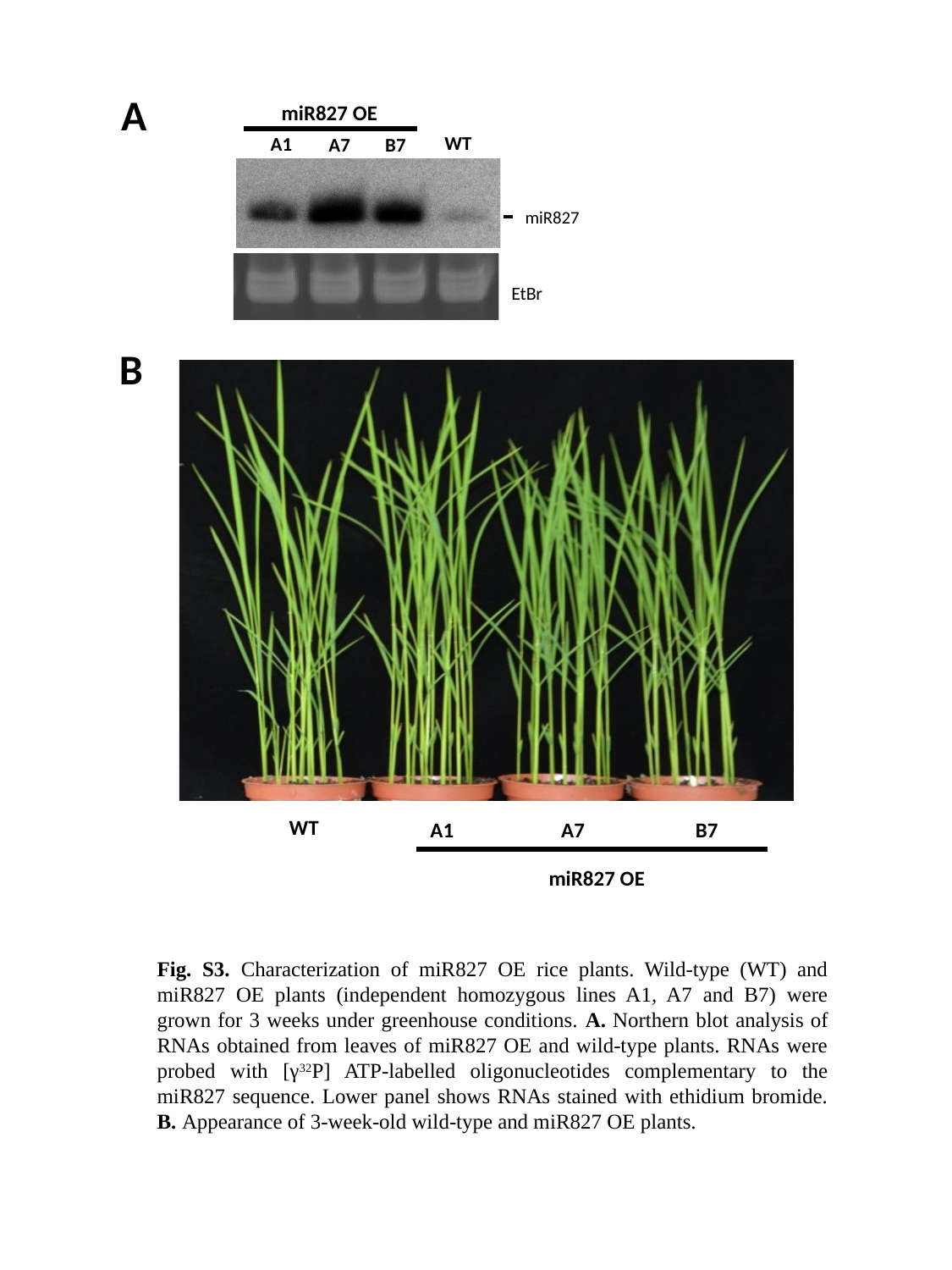

A
miR827 OE
WT
A1
A7
B7
miR827
EtBr
B
WT
B7
A1
A7
miR827 OE
Fig. S3. Characterization of miR827 OE rice plants. Wild-type (WT) and miR827 OE plants (independent homozygous lines A1, A7 and B7) were grown for 3 weeks under greenhouse conditions. A. Northern blot analysis of RNAs obtained from leaves of miR827 OE and wild-type plants. RNAs were probed with [γ32P] ATP-labelled oligonucleotides complementary to the miR827 sequence. Lower panel shows RNAs stained with ethidium bromide. B. Appearance of 3-week-old wild-type and miR827 OE plants.
