# Supplementary material for: Silencing Osa-miR827 via CRISPR/Cas9 protects rice against the blast fungus Magnaporthe oryzae
Source: Plant Mol Biol. 2024 Sep 24;114(5):105. doi: 10.1007/s11103-024-01496-z (PMC11422438; doi:10.1007/s11103-024-01496-z)
Supplement: Supplementary file 4 — Supplementary file4 (PPTX 396 KB) Predicted secondary structure of the miR827 precursor, wild-type (WT) and mutant alleles generated by CRISPR/Cas9 mutagenesis. The fold-back structure of the mutated miR827 precursor was obtained using the RNAfold Web Server (http://rna.tbi.univie.ac.at/cgi-bin/RNAWebSuite/RNAfold.cgihttp://rna.tbi.univie.ac.at/cgi-bin/RNAWebSuite/RNAfold.cgi). The location of the mature miR827 in the precursor structure is indicated with a black line. Colors indicate the base pairing probability. A. Predicted precursor structure of the wild-type and mutant miR827 precursors based on the nucleotide sequence currently annotated in miRBase. B. Precursor structure predicted for the wild-type miR827 precursor upon extending its nucleotide sequence from the genomic region surrounding the MIR827 locus (20 nucleotides upstream/downstream) [file 11103_2024_1496_MOESM4_ESM.pptx]

## Slide 1
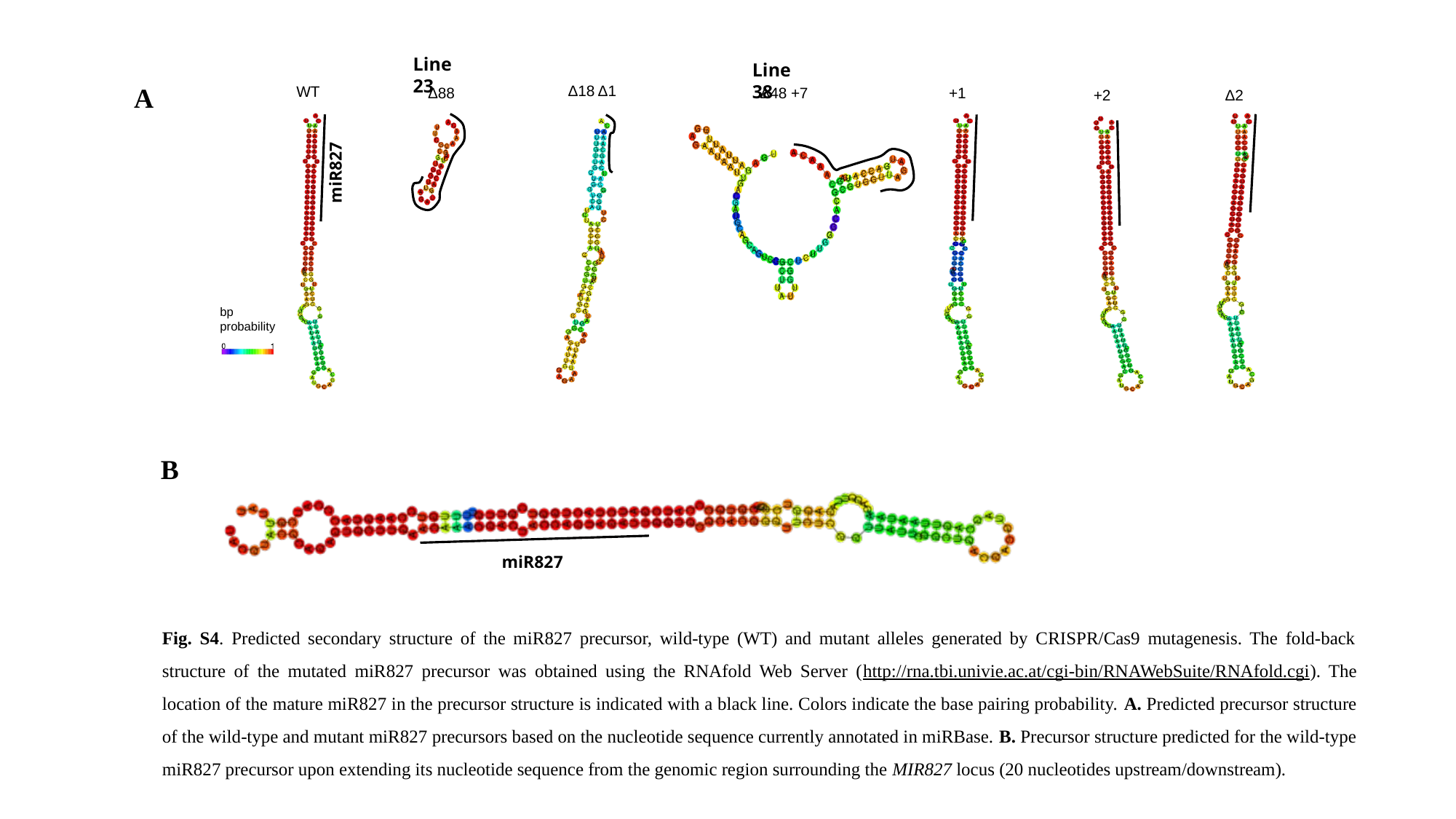

Line 23
Line 38
A
Δ18 Δ1
WT
Δ88
Δ48 +7
+1
+2
Δ2
miR827
bp probability
miR827
B
Fig. S4. Predicted secondary structure of the miR827 precursor, wild-type (WT) and mutant alleles generated by CRISPR/Cas9 mutagenesis. The fold-back structure of the mutated miR827 precursor was obtained using the RNAfold Web Server (http://rna.tbi.univie.ac.at/cgi-bin/RNAWebSuite/RNAfold.cgi). The location of the mature miR827 in the precursor structure is indicated with a black line. Colors indicate the base pairing probability. A. Predicted precursor structure of the wild-type and mutant miR827 precursors based on the nucleotide sequence currently annotated in miRBase. B. Precursor structure predicted for the wild-type miR827 precursor upon extending its nucleotide sequence from the genomic region surrounding the MIR827 locus (20 nucleotides upstream/downstream).
