# Supplementary material for: Silencing Osa-miR827 via CRISPR/Cas9 protects rice against the blast fungus Magnaporthe oryzae
Source: Plant Mol Biol. 2024 Sep 24;114(5):105. doi: 10.1007/s11103-024-01496-z (PMC11422438; doi:10.1007/s11103-024-01496-z)
Supplement: Supplementary file 5 — Supplementary file5 (PPTX 660 KB) Appearance of CRISPR-miR827 rice plants and expression analysis of OsPHR4 and OsSPX-MFS3 in CRISPR-miR827 plants. A. Phenotype of CRISPR-miR827 (lines 23.5 and 38.7) and wild-type (WT, e.g. azygous plants segregated from heterozygous plants) plants. Rice plants were grown under Pi-limiting conditions (Low-Pi) or Pi sufficient conditions for 3 weeks. B. OsPHR4 expression in wild-type and CRISPR-miR827 rice plants grown under Low-Pi supply (black bars) and normal (white bars) Pi conditions. C. OsSPX-MFS3 expression in CRISPR-miR827 plants was determined by RT-qPCR. Data from one representative experiment of three independent experiments are presented, each experiment consisting of a pool of 3 leaves (ANOVA test, * P ≤ 0.05; ** P ≤ 0.01; *** P ≤ 0.001; ns, not significant) [file 11103_2024_1496_MOESM5_ESM.pptx]

## Slide 1
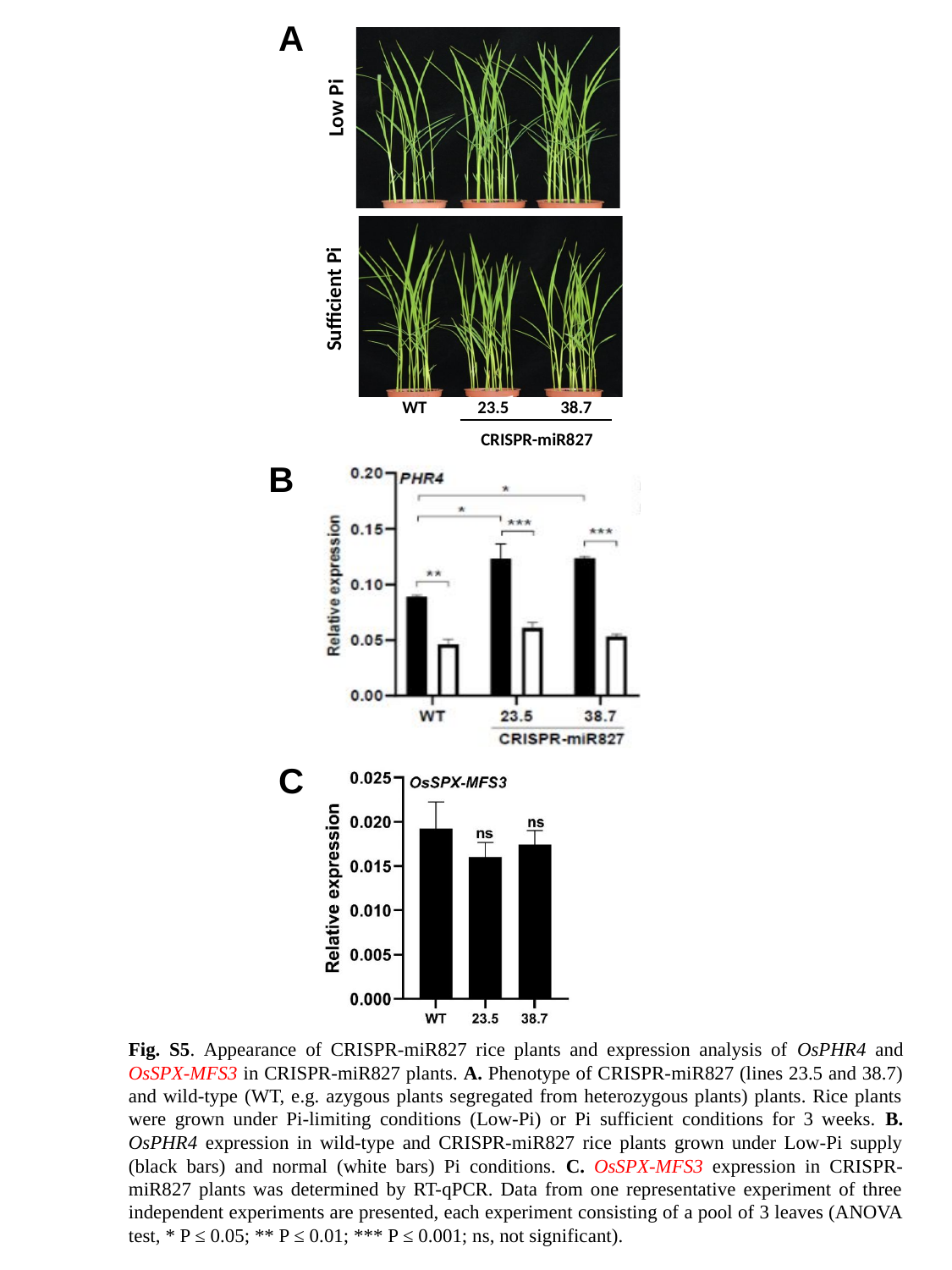

A
38.7
WT
23.5
CRISPR-miR827
Low Pi
Sufficient Pi
B
C
Fig. S5. Appearance of CRISPR-miR827 rice plants and expression analysis of OsPHR4 and OsSPX-MFS3 in CRISPR-miR827 plants. A. Phenotype of CRISPR-miR827 (lines 23.5 and 38.7) and wild-type (WT, e.g. azygous plants segregated from heterozygous plants) plants. Rice plants were grown under Pi-limiting conditions (Low-Pi) or Pi sufficient conditions for 3 weeks. B. OsPHR4 expression in wild-type and CRISPR-miR827 rice plants grown under Low-Pi supply (black bars) and normal (white bars) Pi conditions. C. OsSPX-MFS3 expression in CRISPR-miR827 plants was determined by RT-qPCR. Data from one representative experiment of three independent experiments are presented, each experiment consisting of a pool of 3 leaves (ANOVA test, * P ≤ 0.05; ** P ≤ 0.01; *** P ≤ 0.001; ns, not significant).
