# Supplementary material for: Silencing Osa-miR827 via CRISPR/Cas9 protects rice against the blast fungus Magnaporthe oryzae
Source: Plant Mol Biol. 2024 Sep 24;114(5):105. doi: 10.1007/s11103-024-01496-z (PMC11422438; doi:10.1007/s11103-024-01496-z)
Supplement: Supplementary file 6 — Supplementary file6 (PPTX 70 KB) Pi content in leaves of wild-type, miR827 OE (upper panel) and CRISPR-miR827 (lower panel) plants that have been mock-inoculated (-) or inoculated with M. oryzae spores (+). Pi content was assessed in leaves at two different positions of the same plant, Leaf 2 and Leaf 3, at 24 hours post-inoculation (hpi). Results shown correspond to Leaf 2 (similar results were obtained on leaves at position 3). Bars represent mean ± SEM of 4 biological replicates, each one from a pool of 4 leaves (leaf 2) from independent plants (Two-way ANOVA followed by Tukey’s HSD test). Letters indicate significant differences among conditions [file 11103_2024_1496_MOESM6_ESM.pptx]

## Slide 1
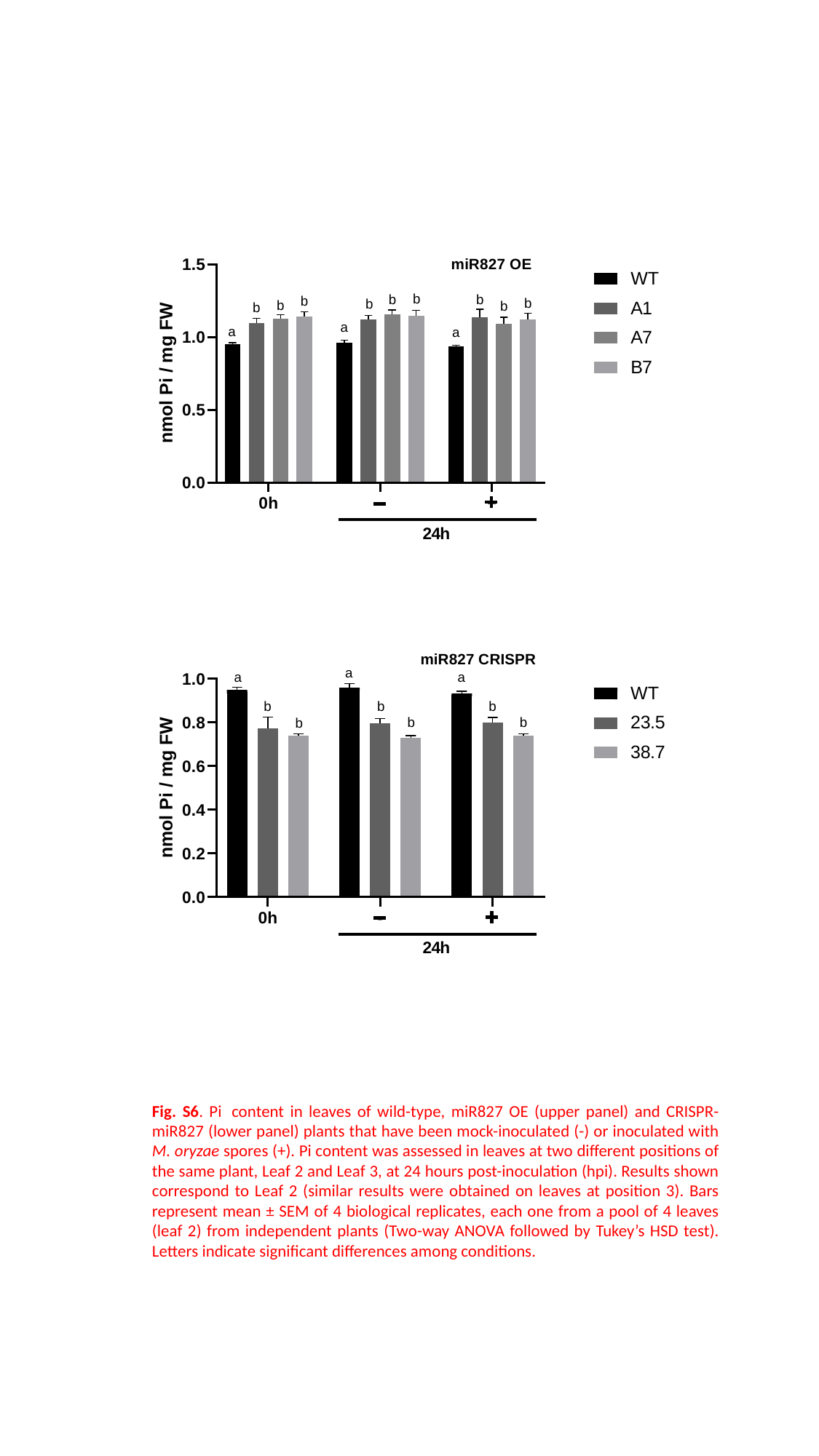

Fig. S6. Pi  content in leaves of wild-type, miR827 OE (upper panel) and CRISPR-miR827 (lower panel) plants that have been mock-inoculated (-) or inoculated with M. oryzae spores (+). Pi content was assessed in leaves at two different positions of the same plant, Leaf 2 and Leaf 3, at 24 hours post-inoculation (hpi). Results shown correspond to Leaf 2 (similar results were obtained on leaves at position 3). Bars represent mean ± SEM of 4 biological replicates, each one from a pool of 4 leaves (leaf 2) from independent plants (Two-way ANOVA followed by Tukey’s HSD test). Letters indicate significant differences among conditions.
