# Supplementary material for: Silencing Osa-miR827 via CRISPR/Cas9 protects rice against the blast fungus Magnaporthe oryzae
Source: Plant Mol Biol. 2024 Sep 24;114(5):105. doi: 10.1007/s11103-024-01496-z (PMC11422438; doi:10.1007/s11103-024-01496-z)
Supplement: Supplementary file 8 — Supplementary file8 (DOCX 19 KB) Prediction of off-targets for gRNAs used to generate mutations in the MIR827 locus. The CRISPR-P 2.0 software designed for genome editing was used for off-target prediction (http://crispr.hzau.edu.cn/CRISPR2/). The scoring system in the CRISPR-P 2.0 tool identifies the most similar sequences with a limited number of mismatches (typically ≤ 3) in comparison to the designed gRNA. The off-target score ranges from 0 to 1, with a higher score denoting targets with lower target potential. The top 20 predicted off-targets are shown. PAM sequences are highlighted in green. Mismatches are indicated in red [file 11103_2024_1496_MOESM8_ESM.docx]

**Table S2.** Prediction of off-targets for gRNAs used to generate mutations in the *MIR827* locus. The CRISPR-P 2.0 software designed for genome editing was used for off-target prediction (<http://crispr.hzau.edu.cn/CRISPR2/>). The scoring system in the CRISPR-P 2.0 tool identifies the most similar sequences with a limited number of mismatches (typically ≤ 3) in comparison to the designed gRNA. The off-target score ranges from 0 to 1, with a higher score denoting targets with lower target potential. The top 10 predicted off-targets are shown. PAM sequences are highlighted in green. Mismatches are indicated in red.

| **sgRNA 1** | | | | | |
| --- | --- | --- | --- | --- | --- |
| **Target site** | TATTGGCTCTTGGGCACGCG**TGG** | | | | |
| **Predicted off-target site** | **Off-target score** | **Mismatches**  **(Ner)** | **Chromosome** | **Gene ID** | **Genome location** |
| **A**ATT**A**GCTCTTGG**A**CAC**T**CG**AGG** | 0,433 | 4 MMs | Chr3:+9346828 |  | Intergenic |
| TATTG**C**CTC**C**TGGGCACG**T**G**GGG** | 0,168 | 3 MMs | Chr4:+22056422 |  | Intergenic |
| **GCG**TGGCTCTTG**C**GCACGCG**CGG** | 0,158 | 4 MMs | Chr6:-22482631 |  | Intergenic |
| TAT**G**GGCT**G**TTGGGCACG**AAGGG** | 0,155 | 4 MMs | Chr6:-28037171 |  | Intergenic |
| T**GG**TGG**T**TCTTGGGCA**A**GCG**AGG** | 0,152 | 4 MMs | Chr3:+4200715 | LOC_Os03g08250 | utr |
| TATTGGC**AG**TT**T**GGCAC**A**CG**GGG** | 0,132 | 4 MMs | Chr8:-24865941 | LOC_Os08g39350 | utr |
| TATT**T**GCTCTTG**C**GCAC**T**C**TGGG** | 0,059 | 4 MMs | Chr12:+18910707 | LOC_Os12g31440 | CDS |
| T**G**TTGGC**G**CT**CC**GGCACGCG**TAG** | 0,054 | 4 MMs | Chr9:-22303523 | LOC_Os09g38830 | CDS |
| T**G**TTGGC**G**CT**CC**GGCACGCG**TAG** | 0,054 | 4 MMs | Chr2:+34511831 | LOC_Os02g56370 | CDS |
| TATTGGCT**A**TTGG**A**CA**G**G**A**G**AGG** | 0,016 | 4 MMs | Chr5:+14507016 |  | Intergenic |
|  | | | | | |
| **sgRNA2** | | | | | |
| **Target site** | TAGATGACCAGCAACAAAAC**AGG** | | | | |
| **Predicted off-target site** | **Off-target score** | **Mismatches**  **(Ner)** | **Chromosome** | **Gene ID** | **Genome location** |
| **GC**GATGACC**T**GCA**G**CAAAAC**AGG** | 0.309 | 4 MMs | Chr5:-26924072 |  | Intergenic |
| **C**A**C**AT**T**ACCAG**A**AACAAAAC**CGG** | 0.157 | 4 MMs | Chr3:+13260808 |  | Intergenic |
| **C**A**A**ATG**T**CC**G**GCAACAAAAC**TGG** | 0.156 | 4 MMs | Chr9:-2010250 | LOC_Os09g03939 | intron |
| T**C**GAT**C**A**G**CA**T**CAACAAAAC**CGG** | 0.132 | 4 MMs | Chr9:+9128339 |  | Intergenic |
| **C**AGAT**C**ACCAG**A**AACAAAA**TGGG** | 0.125 | 4 MMs | Chr8:+741003 | LOC_Os08g02190 | utr |
| **G**AGATGA**TG**AGCAACAA**C**AC**CGG** | 0.099 | 4 MMs | Chr9:+14567948 | LOC_Os09g24480 | CDS |
| TAG**C**T**A**AC**T**AGC**T**ACAAAAC**AGG** | 0.093 | 4 MMs | Chr11:-14322304 |  | Intergenic |
| TAG**G**TG**C**CC**T**GC**T**ACAAAAC**AGG** | 0.072 | 4 MMs | Chr10:-1420157 |  | Intergenic |
| TAGATG**C**C**T**AGCAACA**G**AAC**TGG** | 0.068 | 3 MMs | Chr1:+31797049 | LOC_Os01g55240 | utr |
| TA**A**ATGACCA**A**CA**C**CAAA**G**C**AGG** | 0.060 | 4 MMs | Chr1:+31167681 | LOC_Os01g54190 | utr |
